# Reconsidering the developmental origins of adult disease paradigm: The ‘metabolic coordination of childbirth’ hypothesis

**DOI:** 10.1093/emph/eoae002

**Published:** 2024-01-18

**Authors:** Jonathan C K Wells, Gernot Desoye, David A Leon

**Affiliations:** Population, Policy and Practice Research and Teaching Department, UCL Great Ormond Street Institute of Child Health, 30 Guilford Street, London WC1N 1EH, UK; Department of Obstetrics and Gynaecology, Medical University of Graz, Auenbruggerplatz 14, 8036 Graz, Austria; Faculty of Epidemiology and Population Health, London School of Hygiene and Tropical Medicine, Keppel Street, London WC1E 7HT, UK

**Keywords:** obstructed labour, foetal growth, noncommunicable disease, gestation, genetic conflict, DOHaD hypothesis

## Abstract

In uncomplicated pregnancies, birthweight is inversely associated with adult non-communicable disease (NCD) risk. One proposed mechanism is maternal malnutrition during pregnancy. Another explanation is that shared genes link birthweight with NCDs. Both hypotheses are supported, but evolutionary perspectives address only the environmental pathway. We propose that genetic and environmental associations of birthweight with NCD risk reflect coordinated regulatory systems between mother and foetus, that evolved to reduce risks of obstructed labour. First, the foetus must tailor its growth to maternal metabolic signals, as it cannot predict the size of the birth canal from its own genome. Second, we predict that maternal alleles that promote placental nutrient supply have been selected to constrain foetal growth and gestation length when fetally expressed. Conversely, maternal alleles that increase birth canal size have been selected to promote foetal growth and gestation when fetally expressed. Evidence supports these hypotheses. These regulatory mechanisms may have undergone powerful selection as hominin neonates evolved larger size and encephalisation, since every mother is at risk of gestating a baby excessively for her pelvis. Our perspective can explain the inverse association of birthweight with NCD risk across most of the birthweight range: any constraint of birthweight, through plastic or genetic mechanisms, may reduce the capacity for homeostasis and increase NCD susceptibility. However, maternal obesity and diabetes can overwhelm this coordination system, challenging vaginal delivery while increasing offspring NCD risk. We argue that selection on viable vaginal delivery played an over-arching role in shaping the association of birthweight with NCD risk.

## INTRODUCTION

From the 1980s, adult non-communicable diseases (NCDs) were approached using a new life-course perspective. Going beyond the heritability of these diseases and their well-known associations with adult lifestyle, epidemiological studies broke new ground by linking variability in birthweight with the risk of cardiovascular disease, hypertension and type 2 diabetes (T2DM) many decades later [1–3]. This indicated a vital contribution of development *in utero*.

The resulting ‘developmental origins of adult health and disease’ (DOHaD) paradigm [4], attributing a component of NCD risk to early developmental experience, has become influential in both biomedical research and public health. Experimental research on animals confirms that manipulation of maternal nutrition during pregnancy causes metabolic abnormalities in the offspring, with implications for adult disease risk [5]. The science of epigenetics offers insight into underlying mechanisms [4]. Moreover, birthweight, even if not on the causal pathway to disease, is a robust predictor of adult NCDs [6].

Initially, most attention focussed on the elevated NCD risk associated with low birthweight (<2500 g) and the likely mechanism of maternal undernutrition before or during pregnancy [1, 3]. However, for uncomplicated pregnancies, the association with NCDs is not merely elevated among those with low birthweight, but declines in dose–response manner across the normal birthweight range [3, 7, 8], suggesting that severe maternal undernutrition is not the primary underlying mechanism. Moreover, higher birthweight (>4500 g) is also associated with elevated NCD risk, especially in females [6, 9], which may relate to maternal gestational diabetes and its association with excess foetal adiposity. A meta-analysis of 135 studies found that risks for adult T2DM, cardiovascular disease and hypertension were lowest for relatively high birthweights of 3.5–4.0, 4.0–4.5 and 4.0–4.5 kg, respectively [6]. There is thus compelling evidence that birthweight predicts adult NCD risk, but the nature of the association is non-linear.

Published in 1999, the alternative ‘foetal insulin’ hypothesis proposed that genes underlie the link between poor foetal growth and adult T2DM risk [10]. Initially, evidence from monogenic disorders of insulin metabolism and genome-wide association studies supported the notion of a shared genetic predisposition to lower birthweight and adult NCDs [11–13]. Subsequent work showed that the foetal insulin hypothesis also applies to high birthweight, as mutations that promote macrosomia via increased foetal insulin secretion are associated with diabetes in adulthood [14].

Nevertheless, environmental pathways cannot be discounted as, among monozygotic twins, the twin with lower birthweight has elevated NCD risk [15]. The DOHaD and foetal insulin hypotheses therefore appear complementary, whereby the life-course aetiology of NCDs, reflecting a reverse J-shaped association of birthweight with adult disease risk, has both genetic and environmental components. Currently, a comprehensive conceptual framework linking these approaches is lacking.

To understand *why* adult morbidity and mortality are correlated with foetal growth requires an evolutionary perspective. For the DOHaD framework, Hales and Barker offered one such perspective in their ‘thrifty phenotype’ hypothesis: this conceptual approach assumed that to protect their own survival and reproductive potential, undernourished mothers reduce placental nutrient supply and hence the birthweight of their offspring [16]. Following this adjustment, the surviving offspring suffers elevated NCD susceptibility in later life [16]. An alternative perspective is the ‘Predictive Adaptive Response’ hypothesis, which assumes that exposure to maternal undernutrition during pregnancy drives the foetus not only to reduce its immediate growth but also to adjust its metabolic phenotype in anticipation of experiencing similar undernutrition in adulthood [17]. Traits such as insulin resistance and central body fat are assumed to be triggered during foetal development in order to promote survival and reproduction in energy-scarce adult environments. However, the validity of the PAR hypothesis for long-lived species such as humans has been challenged on both theoretical [18, 19] and empirical [20, 21] grounds: in particular, signals of maternal nutritional supply during pregnancy are unlikely to be predictive of the ecological conditions that will be experienced throughout the adult reproductive career [22].

Crucially, an adaptive perspective for the genetic association of birthweight with adult NCDs remains lacking. If higher birthweights predict both better survival of infants after birth [23], the life-course period with the greatest mortality, and lower adult NCD risk [24], why would alleles countering both these benefits evolve? More broadly, why are the majority of birthweights lower than those that both maximize neonatal survival and minimize adult NCD risk [6, 23, 25]? Here, we propose a unifying framework for the DOHaD and foetal insulin hypotheses, focussed on genetic and physiological mechanisms that reduce the risk of obstructed labour.

## THE ‘METABOLIC COORDINATION OF CHILDBIRTH’ HYPOTHESIS

Both mother and offspring are exposed to a range of mortality risks around the time of childbirth [26, 27]. Obstructed labour *sensu lato* refers specifically to the risk that the baby is too big to pass down the birth canal, which can impact maternal and neonatal mortality through several different mechanisms [28]. We propose that, since both mother and foetus are exposed to mortality risk from this source [29], both parties may evolve genetic and physiological strategies to reduce the risk. Both parties gain Darwinian fitness advantages from increased foetal growth because birth size is a robust predictor of early survival [30–32] and may also benefit the offspring’s reproductive fitness [33]. However, without a counterbalancing regulatory system, the foetus may grow too large for vaginal delivery.

Our conceptual approach leads to two broad predictions about the relationship of alleles with growth and NCD risk. First, we predict a relatively low influence of foetal genotype on birthweight. While the dimensions of the obstetric pelvis are partly heritable [34], they are potentially susceptible to constraint during maternal development [35]. Shorter women who experienced poor growth have reduced pelvic dimensions and an increased risk of obstructed labour [36–38]. Accordingly, the foetus cannot interrogate its own genotype to predict the dimensions of its mother’s birth canal and must therefore adjust its prenatal growth trajectory to maternal metabolic or epigenetic signals [39].

Second, we propose that maternal and foetal alleles may contribute to a co-adaptive regulatory system, whereby alleles expressed in the mother that raise the capacity for nutritional investment may, when expressed in the foetus, restrain its growth response. Conversely, alleles expressed in the mother that promote dimensions of the birth canal, thereby reducing risk of obstructed labour, may also promote foetal growth. Through these mechanisms, we argue, the growth of each foetus is tailored to the skeletal capacity of its mother to deliver it.

**Figure 1.**
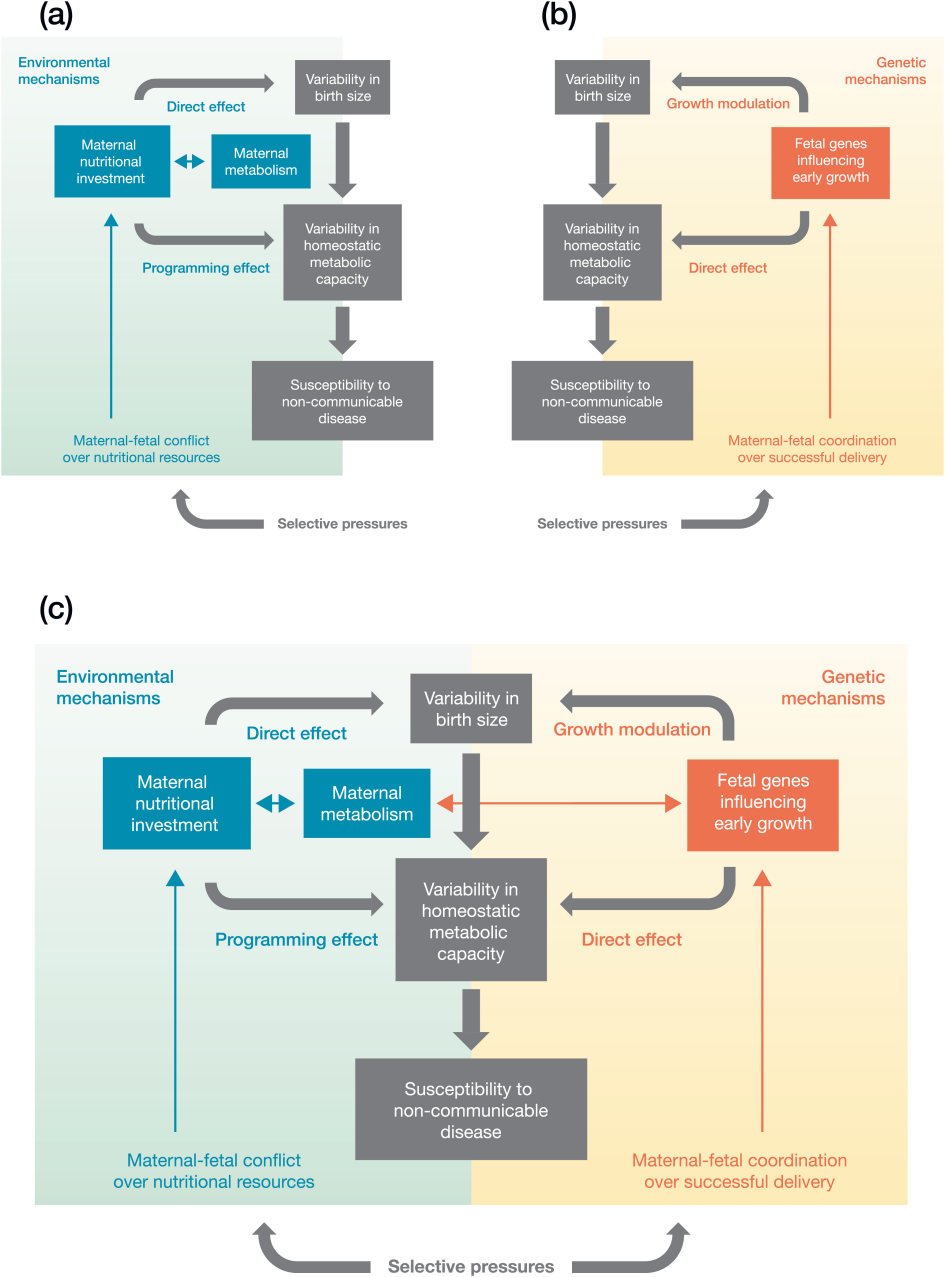
Contrasting conceptual models of the role of birthweight in the developmental origins of adult non-communicable disease (NCD). Green text and arrows represent environmental mechanisms (phenotypic plasticity), while red text and arrows represent genetic mechanisms. Upper panel: (**a**) The environmental model describing the ‘thrifty phenotype’ hypothesis, which assumes that maternal malnutrition in pregnancy reduces birthweight, and that the surviving offspring therefore has elevated susceptibility to NCDs. The long-term effects of maternal phenotype on offspring NCD risk are sometimes termed ‘programming effects’. The underlying selective pressure is assumed to be maternal-foetal conflict over metabolic resources in pregnancy. (**b**) The genetic model describing the ‘foetal’ insulin hypothesis’, which assumes that common alleles contribute to low or high birthweight, and also to the associated adult NCD susceptibility. No selective pressure has been proposed for this hypothesis. Lower panel: (**c**) The combined model, which extends the version presented by Hatterlsey and Tooke in the foetal insulin hypothesis [10]. The combined model includes obstructed labour as another selective pressure that underlies both genetic and environmental associations of birthweight with NCDs, and assumes that foetal genes can both impact and respond to maternal metabolism.

We therefore propose that selection has favoured a composite system of physiological signals and alleles regulating foetal size and gestation length (**Fig. 1**). Addressing both gestation length and birth size is central to our argument, as around a quarter of foetal weight at 38 weeks typically accumulates in the preceding 3 weeks [40]. Reducing the length of gestation is one potential mechanism that allows a foetus with high growth potential to be delivered.

The cost of this regulatory system is that the mechanisms that protect the mother and foetus against obstructed labour may also elevate the offspring’s susceptibility to adult NCDs. According to the ‘capacity-load’ life-course model of NCDs [41], any mechanism (whether genetic or facultative) that reduces birthweight may also undermine the long-term metabolic capacity for homeostasis that protects against ageing and NCDs. Birthweight is a composite marker of muscle and organ development *in utero* and shows multiple dose-response associations with organ phenotype [41–46], and hence the capacity for homeostasis in adult life [47–49]. Whether or not those born smaller do develop overt disease, however, depends strongly on adult phenotype. In contemporary settings, the association of lower birthweight with NCD risk is amplified by exposure to high levels of ‘metabolic load’ in adulthood, relating to factors such as obesity, unhealthy diet, sedentary behaviour and smoking [41, 50, 51].

## OBSTRUCTED LABOUR AS A SELECTIVE PRESSURE

The evolutionary pressure of obstructed labour in the hominin lineage was first highlighted by Washburn [52], building on work by Krogman [53]. Washburn suggested that the dimensions of the human birth canal had been exposed to antagonistic selective pressures associated with the emergence of bipedal posture and encephalised neonates. His interpretation of this ‘obstetrical dilemma’ was that it resulted in human offspring being born at a relatively early stage of development. Subsequent work has shown that our gestation is not short compared to other apes [54, 55], while there is discussion over whether maternal energetic limits might also explain the typical duration of human pregnancy [55, 56]. Nonetheless, maternal pelvic dimensions may still be subject to evolutionary constraint [57]. For example, the dimensions of the anteroposterior oval outlet and inlet are considered to have responded to contrasting selective pressures, associated with pelvic floor support and upright posture, respectively [58, 59].

Moreover, high levels of maternal mortality in many populations, partly due to obstructed labour [29], confirm that human childbirth is indeed risky. In Ethiopia, for example, approximately one of every seven maternal deaths has been attributed to obstructed labour [28], and even where it does not cause mortality, it may inflict other forms of morbidity on either the mother (e.g. obstetric fistula, uterine rupture) or offspring (e.g. neonatal asphyxia) that may substantially reduce the fitness of each party. Among 4396 women from nine sub-Saharan African countries presenting with obstetric fistula for repair, for example, 84% had previously delivered a stillborn baby [60]. Untreated by surgery, fistula is likely to terminate a woman’s reproductive career.

Inadequate medical services in such settings contribute to the high mortality and morbidity burden associated with obstructed labour [32], but its risk is also systematically associated with maternal physical traits. In diverse populations, maternal short stature is associated with an increased risk of obstructed labour and birth injuries such as fistula [61–63]. Other studies have linked obstructed labour with narrower midplane medio-lateral pelvic dimensions and with contraction in the anterior-posterior dimension [64, 65]. Importantly, the risks associated with smaller pelvic dimensions further depend on the weight and head size of the neonate [36, 37].

The ‘cliff edge’ hypothesis offers another perspective on obstructed labour, by assuming that childbirth is characterized by the interaction of contrasting fitness functions [66]. The discrepancy between pelvic and foetal dimensions demonstrates a normal distribution, whereas individual female fitness is characterized by a ‘cliff-edge’ form, where passage through the birth canal becomes impossible once foetal size exceeds a threshold [66]. On this basis, the phenotypic distribution that maximizes population mean fitness is inevitably associated with a proportion of foetuses being too large for natural delivery. We argue that this scenario would maintain selective pressure on mechanisms that coordinate foetal and maternal dimensions to reduce obstructed labour risk.

Given that both maternal and foetal skeletal traits are relevant to obstructed labour, the risk of childbirth complications may appear to be fundamentally an anatomical issue. In the immediate term, for example, obstructed labour is solved by surgery (caesarean section, symphysiotomy, episiotomy) or by instrumental delivery (forceps, vacuum extraction), though these procedures have their own risks to health and wellbeing [28].

We propose a different perspective: incorporating a metabolic perspective on childbirth allows it to be conceptualized as a ‘coordination problem’ [67]. Specifically, we propose that selection has favoured the coordination of maternal and foetal metabolic interactions over foetal growth and gestation in ways that optimize the compromise over maximizing the fitness of each party, which includes the joint interest of minimizing obstructed labour risk.

## MATERNAL SIGNALS AND FOETAL GROWTH

Over evolutionary time, the threat of obstructed labour might be resolved by the ‘genetic coordination’ of maternal and foetal size, whereby their shared alleles result in each foetus expressing a magnitude of growth appropriate for its mother’s capacity for delivery. On average, taller mothers have larger birth canals and can deliver larger foetuses [68–70], but the specific role of alleles in these associations remains unclear.

Adult size (notably stature) is highly heritable [71], but little is known about maternal pelvic dimensions. In a study comparing 30 monozygotic and 30 dizygotic twin pairs from India, 60–80% of the variability in pelvic traits could be attributed to genetic factors [34]. However, these estimates may be unreliable as the data invalidated some of the assumptions underlying the classic twin study design [54]. The association of maternal height alleles and pelvic dimensions is also unknown.

Several studies have provided suggestive evidence of genetic co-regulation of foetal growth in association with maternal skeletal size. Among Indian adolescents and adults, taller women and those with broader shoulders tended also to have larger pelvic dimensions [72]. Another study of US skeletons found that women with large head dimensions had a birth canal shaped favourably for delivering large-headed neonates [73]. Despite these intra-individual correlations, however, whether maternal pelvic dimensions are directly associated with those of the neonate through genetic mechanisms has not been assessed.

Moreover, there are several reasons why the foetus cannot interrogate its own genome to determine how large it should grow, while being able to pass down the birth canal. First, exposure to adverse conditions in early life (e.g. ecological shocks, undernutrition, high burden of infections) may prevent women from achieving their genetic potential for pelvic growth. Stunting in childhood remains a highly prevalent issue in low- and middle-income countries [74], and growth failure in the first year of life is strongly associated with short stature in adulthood [75]. We assume that throughout human evolution, reducing the allocation of energy to growth was a key component of developmental adaptation to energy scarcity [76]. Second, each foetus shares only 50% of its genes with its mother; hence any paternal genetic influence could drive higher levels of foetal growth than would permit safe delivery. There is evidence that both of these mechanisms increase the risk of obstructed labour.

**Figure 2.**
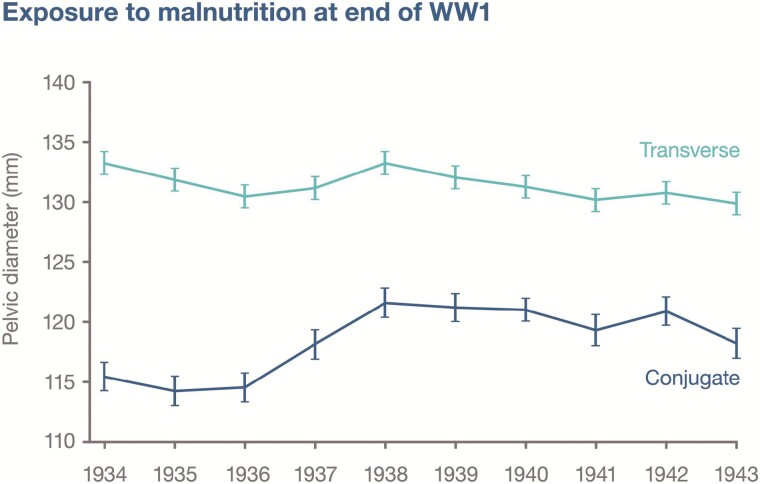
Variability in mean conjugate and transverse diameters of the pelvis, obtained by radiology, in 640 primigravid women presenting for delivery in Moreton-in-Marsh District Hospital, UK between 1934 and 1943. Mean length of the conjugate diameter was significantly reduced among women who were likely to have been exposed during early childhood to malnutrition at the end of World War 1, whereas mean length of the transverse diameter was relatively unaffected. Based on data from Nicholson [78].

First, the impact of childhood undernutrition on the obstetric pelvis was already recognized in the late 19th century. In the UK, the majority of caesarean sections in this period were attributed to rickets, which deforms the shape of the birth canal [77]. Less severe malnutrition may also reduce some dimensions of the pelvis. **Figure 2** illustrates a 7 mm (~6%) deficit in the average conjugate diameter of women delivering in a UK hospital in the mid-1930s compared to subsequent years [78]. The author attributed this difference, which was not replicated for the transverse diameter, to early childhood exposure to undernutrition at the end of World War 1, when food intake was substantially constrained by the combination of high prices, harvest failures and a maritime blockade [79]. Even if his explanation is incorrect, the data clearly indicate the impact of some kind of ecological stress on pelvic dimensions. Shorter tibia length, a marker of childhood growth that is especially sensitive to environmental influences [80], has been associated with smaller dimensions of the obstetric pelvis in nulliparous South Asian women [69].

Second, the possibility that paternal alleles may drive foetal growth beyond the level compatible with safe delivery is demonstrated by studies of parents with contrasting body size. In a UK cohort, greater discrepancy in parental heights increased the odds of emergency caesarean section [81]. Likewise, in a study of inter-ethnic unions, the parental combination of Asian mothers (on average, relatively shorter) and white European fathers (on average, taller) was associated with increased odds of caesarean section, compared to same-ethnicity couples with more similar heights [82]. This increased risk may be mediated by birthweight effects, as the pairing of Asian mothers with European fathers results in larger neonates than the pairing of two Asian parents [83–85].

These two mechanisms interact: numerous studies have shown that shorter women have smaller pelvises [68, 86, 87], that place them at increased risk of obstructed labour and emergency caesareans [38, 61, 88]. However, as demonstrated in a Guatemalan cohort, the odds of shorter mothers needing a caesarean further increase in association with larger neonatal head circumference [36]. Similarly, a study in Israel showed that the risk of cephalo-pelvic disproportion was associated with a high neonatal head girth relative to maternal pelvic dimensions [37].

Notably, the component of neonatal size that has been linked with adult NCDs is birthweight, whereas the component most often linked with obstructed labour is neonatal head circumference, as reflected in the term ‘cephalopelvic disproportion’. In Box 1, we show that these neonatal traits are closely associated, indicating that both would be subject to the selective pressure of obstructed labour.

Box 1. **The association of neonatal weight and head circumference, and its significance for childbirth complications**

The DOHaD hypothesis emerged from pioneering studies that linked adult NCD risk with birthweight [1–3, 6]. In contrast, the component of neonatal size most strongly associated with the risk of obstructed labour is the foetal head [89], through high birthweights are also an established risk factor for emergency caesarean section [61, 90]. Detailed studies indicate that the two anthropometric traits are closely related. In an Australian cohort, for example, the correlation of neonatal head circumference and weight was 0.70, while multiple regression analyses confirmed that it was one of the two strongest anthropometric predictors of birthweight alongside chest girth [91].

Similar to birthweight, the influence of foetal alleles on neonatal head circumference appears relatively low [92]. In the Medical Birth Registry of Norway, for example, an analysis of over 77,000 families found that foetal genetic factors explained only 31% and 27% of the variance in birthweight and head circumference, respectively, while maternal genetic factors explained 22% and 19%, respectively [93]. Even though adult head circumference has high heritability [92], the trajectory of foetal head growth, therefore, is primarily determined by maternal metabolic signals.

Since head circumference is less commonly measured than birthweight, its contribution to both short-term and long-term risks may not have been fully appreciated. In an Israeli study of over 26,000 singleton deliveries, large head circumference was a stronger predictor of unplanned caesarean delivery than was high birthweight [94]. Similarly, in their early studies, Barker and colleagues noted inverse associations of adult cardiovascular mortality risk with both birthweight and neonatal head circumference [95]. However, one reason why birthweight may be especially effective in predicting adult NCD risk is that other organs and tissues buffer the foetal brain from nutritional stress, as recognized in the thrifty phenotype hypothesis [16]. Moreover, at higher levels of foetal size, greater weight may encompass many individual traits that impede delivery, including head and abdominal circumferences and shoulder width.

## UNTANGLING THE GENETIC REGULATION OF FOETAL GROWTH

With so many different factors impacting foetal growth, understanding exactly how it is regulated is complex. A new generation of genetic studies is shedding new light, first by improving our ability to discern causal associations of metabolic traits with foetal outcomes and second by providing the capacity to differentiate the effects of foetal versus parental alleles [96].

Recent Mendelian Randomization studies have clarified the parental genetic contributions to birth size. In a study of 3485 mother/infant pairs from Scandinavian birth cohorts, fetally expressed alleles were correlated with birthweight and length, whereas maternal height alleles did not impact birth size through intra-uterine mechanisms [96]. To the extent that the foetus inherits alleles associated with taller maternal height, therefore, it is already larger at birth.

However, studies show that only ~30% of birthweight variance is typically attributed to foetal alleles (Supplementary Table S1). This is consistent with our argument that an unmodulated influence of foetal genotype on birth size would be risky, given that the growth of the maternal pelvis could potentially be disrupted by environmental constraints such as undernutrition or recurrent infections during the mother’s early life [12]. The low heritability of body size through foetal life and infancy compared to later ages (Supplementary Fig. S1), which also applies to head circumference [92, 93], demonstrates the suppression of genetic influence on the foetal growth trajectory and its corresponding sensitivity to environmental (i.e. maternal) influences.

Contrasting with foetal alleles, maternal metabolism is a well-established determinant of foetal growth in mammals in general [97, 98], including humans [99]. Mendelian Randomization studies have associated a maternal haplotype-based genetic score for pre-pregnancy body mass index (BMI) with offspring birthweight, whereas the foetal genetic score was unrelated [100, 101]. Maternal BMI also showed a non-transmitted maternal effect on birthweight [100], while alleles associated with higher maternal fasting plasma glucose were likewise associated with larger offspring birthweight [100, 101]. The findings for plasma glucose clearly demonstrate a causal effect of maternal substrate on foetal growth, but the BMI data merit a more cautious interpretation.

Historically, BMI has been widely used as a proxy for body fatness [102], however, many other components of body size and composition also correlate with BMI [103, 104]. In a Brazilian cohort, the association of maternal BMI with birthweight disappeared once pelvic dimensions were accounted for [105]. Another study of French women linked the maternal conjugate diameter with the suboccipito-bregmatic diameter of the foetal head [106]. The implication is that signals shaping foetal nutrition and growth may be driven by maternal anatomy as well as metabolism. This interpretation is supported by several historical cohort studies that linked reduced maternal pelvic dimensions both with shorter maternal height and with lower birthweight and elevated adult NCD risk in the offspring [70] (Supplementary Table S2). Collectively, this evidence indicates that the pelvic constraint of foetal growth contributes to the inverse association of birthweight with adult NCD risk.

While birth size may be the primary ‘axis of flexibility’ in foetal growth relevant to obstructed labour, gestation length is also relevant. Since birthweight and head circumference increase with gestation, taller mothers who are likely to have larger pelvic dimensions may tolerate longer gestations. Consistent with extensive epidemiological evidence on this issue (Supplementary Table S3) [107], recent Mendelian Randomization studies demonstrated positive relationships of the maternal height genetic score with gestation length [96, 100]. Whereas the height genetic score of both parents was associated with birthweight, neither paternal nor foetal scores were related to gestation length. This indicates a unique causal effect of maternal height on the duration of pregnancy [96] that we argue may be at least partially explained by the positive association of maternal height with pelvic dimensions [68, 69, 108]. Moreover, a meta-analysis of 37 studies found that maternal short stature is a risk factor for spontaneous preterm delivery, suggesting that precocious childbirth might, in part, be triggered to prevent obstructed labour among mothers with reduced height and pelvic dimensions [109].

The evidence reviewed above indicates two key issues. First, while there is some influence of the foetal genotype on growth, the magnitude of the effect is modest, and foetal growth is primarily regulated by maternal metabolic signals. Second, some of these signals relate to maternal physical dimensions, as well as directly to placental fuel transfer, and these signals generate causal effects on both gestation length and foetal size. We now consider in more detail how the mother and foetus interact, to coordinate foetal growth with the mother’s capacity to deliver.

## FOETAL TRADE-OFFS

All other things being equal, any foetal allele from either parent that promotes growth inherently increases obstructed labour risk, particularly for alleles with large magnitude of effect. The hypothesis that selection acts against such large-effect alleles is broadly supported by genome-wide association studies. Although a very small number of fetally expressed alleles have been reported to increase birthweight by up to 90 g [110–112], such large effects are extremely rare and the typical magnitude of effect of ‘birthweight alleles’ is 20–30 g [12, 113]. Importantly, however, such alleles are also associated with shorter gestation, indicating that a foetal trade-off between growth and gestational length ameliorates obstructed labour risk [100].

As highlighted by Haig, some of these growth-promoting alleles generate effects on birth size by actively manipulating maternal metabolism (increasing maternal blood pressure and insulin resistance to force more nutrients across the placenta) while the mother responds with antagonistic signals that constrain nutrient transfer [114]. This ‘tug-of-war’ is assumed to have evolved to help the foetus contest a limited nutrient supply [114].

However, if the mother is well-nourished, or cannot maintain fuel homeostasis, or has alleles raising fasting plasma glucose and blood pressure, foetal size could increase to a degree that increases obstructed labour risk. We propose that selection has acted on the foetal genome to defend against this scenario. Consistent with this prediction, Mendelian Randomization studies show that if alleles promoting maternal fasting plasma glucose are present in the foetus, they are associated with reduced birthweight [100]. The underlying mechanisms require further elucidation, but the existing data indicate a ‘see-saw’ interaction, whereby maternal alleles that upregulate fuel transfer and promote foetal growth are balanced by defensive effects when expressed in the foetus. Paternal insulin resistance is also associated with lower birthweight, suggesting that alleles that may favour weight gain in later life suppress growth when expressed in foetal life [100, 115].

Similar balancing associations are evident for alleles associated with maternal blood pressure and demonstrate effects for both gestation and birth size [100]. Across the normotensive range, higher maternal blood pressure is associated with higher birthweight [116]. However, Mendelian Randomization studies have shown that when fetally expressed, blood pressure alleles of both parents are associated with reduced birthweight [100, 101]. Fetally expressed maternal blood pressure alleles are also associated with shorter gestation [100, 117]. In one study, for example, the foetal expression of alleles associated with a 1 z-score increase in maternal blood pressure was associated with 94 g lower birthweight and 2.3 days shorter gestation [100]. At a mechanistic level, the negative effect of maternal blood pressure alleles on birthweight may be mediated by reduced placental growth [118].

**Figure 3.**
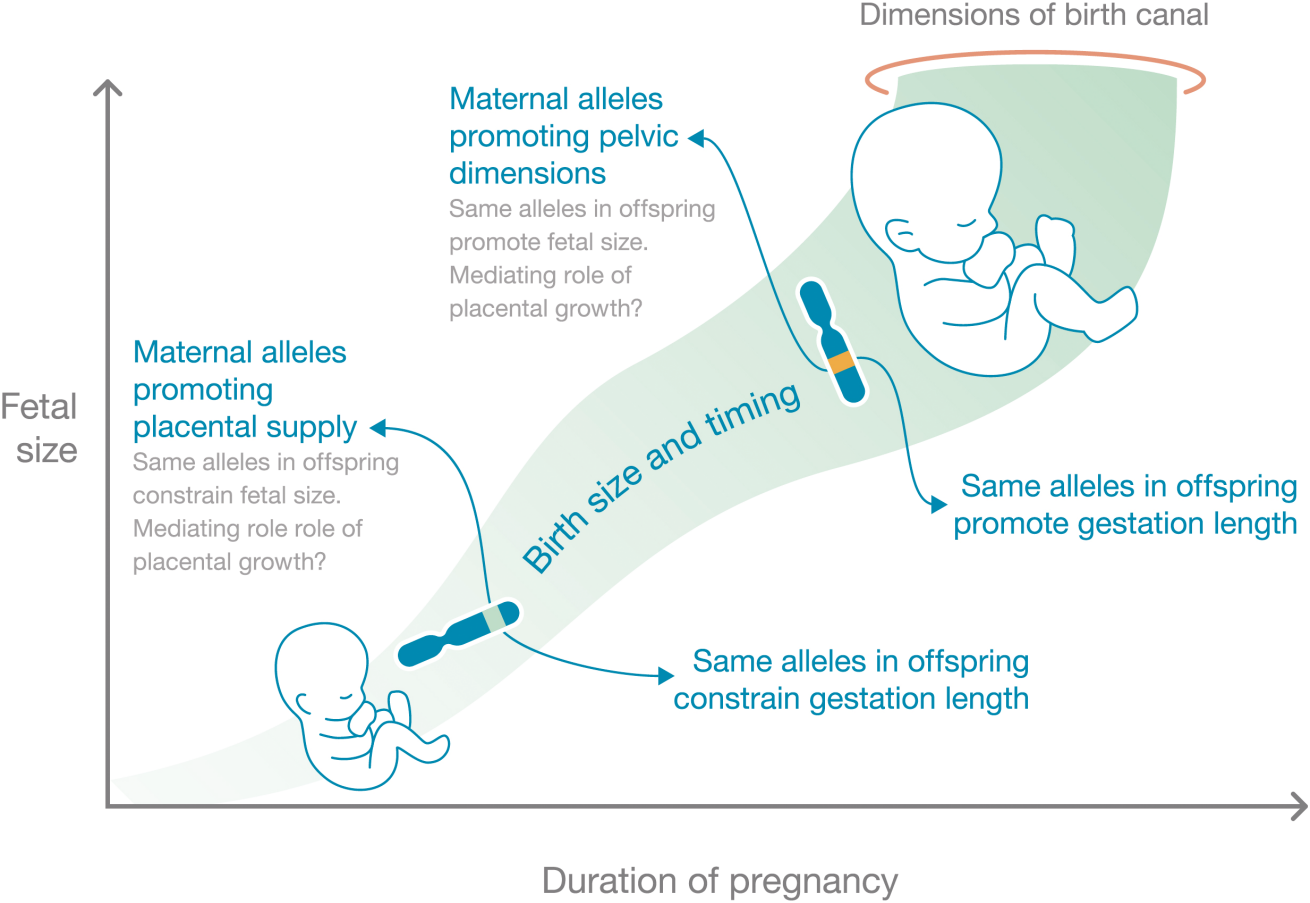
Hypothesized genetic regulatory systems that enable coordination of foetal growth with maternal anatomical capacity for delivery. Maternal alleles may, when expressed in the foetus, either promote or impede foetal growth and gestation length, depending on whether the alleles also promote the size of the maternal birth canal. Variability in placental growth may mediate these interactions.

Collectively, these findings indicate that selection has favoured a metabolic coordination system involving gene expression and function, whereby maternal alleles that raise the capacity to invest in the foetus produce a larger baby but nevertheless reduce the risk of obstructed labour by shortening gestation. If expressed in the foetus, moreover, the same alleles further defend against obstructed labour by reducing both birth size and gestation (**Fig. 3**). These defensive effects might also demonstrate *gene x environment* interactions, and be mediated by components of maternal phenotype such as parity and nutritional status. Accordingly, associations of foetal alleles with birthweight might be different in contemporary compared to ancestral populations.

Among the factors that might impact the selective pressures driving maternal-offspring coordination, foetal sex merits particular attention. On average, boys weigh around 100g more than girls at birth, and also have larger head size [119]. On this basis, for any given size of the maternal pelvis, boys would be predicted to face a riskier delivery compared to their female siblings. That foetal sex influences maternal metabolic health during pregnancy is already well-established [120], but its association with childbirth complications has received less attention. In Box 2, we review evidence for the greater risks of obstructed labour faced by sons and their mothers.

Box 2. **The childbirth risks associated with sons**

Perhaps because it is non-modifiable, foetal sex is rarely considered as an independent risk factor for obstructed labour or its associated mortality and morbidity costs. However, the available evidence demonstrates that sons carry increased risks of adverse outcomes for both themselves and their mothers. Compared to daughters, sons have a higher risk of macrosomia, shoulder dystocia, cephalo-pelvic disproportion and emergency caesarean [121–126]. In addition to caesareans and shoulder dystocia contributing to immediate maternal mortality risk, mothers of sons may also pay long-term costs, relating to their increased likelihood of obstetric fistula [121].

Given these costs, there must be counter-balancing fitness pay-offs to mothers for delivering larger sons. The Trivers-Willard hypothesis assumes that larger sons will achieve disproportionately greater Darwinian fitness compared to larger daughters [127], and that this benefit to maternal inclusive fitness could explain the modest sexual dimorphism in birth size. In contemporary populations, however, maternal obesity and diabetes are amplifying the excess costs of sons, and the high birthweights that disproportionately affect males can often be resolved only by surgical intervention [125].

However, sons might also mitigate these costs, by increased sensitivity to maternal signals. For example, in high-income settings with higher birthweights, males are more likely to be born preterm than females [128], though a range of other factors may also contribute.

Such coordination systems to facilitate childbirth do not discount the possibility of maternal-offspring conflict over nutritional investment, as described by Trivers and Haig [114, 129]. Indeed, the pre-existence of such conflict in hominins may have increased the strength of selection on the mechanisms that reduce the risk of obstructed labour [130], in particular as the *Homo* genus evolved neonates with larger brains and bodies [54]. ‘Arms races’ associated with imprinted genes may also have increased the risk of overshoots in foetal growth (high or low birthweight) [131], with further implications for both obstructed labour and NCD risk. However, in many contemporary settings, the main source of high birthweight is now maternal obesity and diabetes, as discussed further below.

## Implications

Our approach repositions the birthweight-NCD association as a long-term collateral cost of mechanisms that initially benefit maternal and offspring fitness by preventing obstructed labour. The ‘risk’ alleles (higher fasting plasma glucose, blood pressure) are strategies to upregulate maternal investment, potentially selected through ancestral exposure to undernutrition. However, we propose that their expression in mothers with plentiful metabolic substrate, especially among those with impaired pelvic growth, resulted in selection for counterbalancing defence mechanisms that constrain gestation or birthweight. Our perspective has major implications for how we understand the developmental origins of adult disease, and what interventions might help address it.

First, through this lens, the intergenerational transmission of NCD risk can be seen primarily not as a direct consequence of foetal exposure to unhealthy environments, but rather as the by-product of the coordination strategy. The heritability of the ‘trade-off package’ that balances foetal growth against obstructed labour risk results in NCD risk also being heritable, without adult NCDs themselves (typically occurring later in life and impacting fitness through compromising grandparental rather than parental investment [132]) being strongly exposed to selection.

Second, in contrast to much DOHaD research, we place less emphasis on overt maternal undernutrition or pathology as the primary developmental driver of NCDs, and more on stable components of maternal phenotype relevant to childbirth. Undoubtedly, both maternal undernutrition and placental dysfunction, likewise maternal infection, can impact foetal growth [5]. However, there is increasing awareness that these factors may have been given too much emphasis in DOHaD research, and that more stable components of maternal phenotype deserve greater attention [105, 106]. While already recognized in this context on the basis of historical cohort studies (Supplementary Table S2) [70], the obstetric pelvis merits further research.

**Figure 4.**
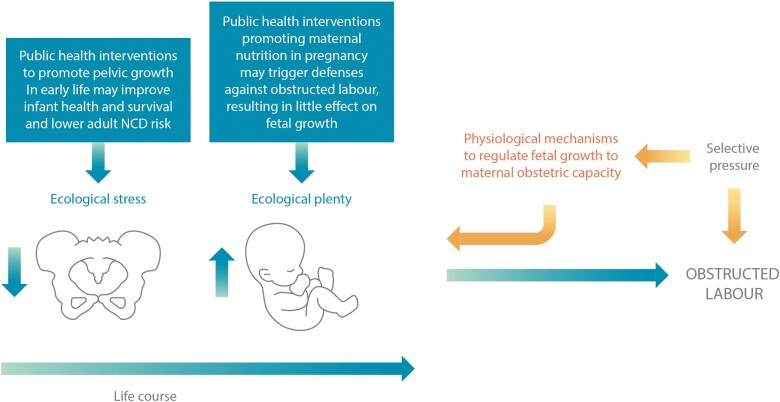
A universal life-course model of the risk of childbirth complications. The risk that the foetus is too large for the mother to deliver may occur in any mother, but particularly when pregnancy phenotype is shaped by better nutritional conditions compared to those experienced by the mother in early life. Selection is expected to favour a universal defence mechanism against this threat. Public health interventions to improve birthweight and reduce adult risk of non-communicable disease (NCDs) may be better targeted at maternal development, to promote pelvic capacity.

Importantly, our hypothesis would explain why birthweight-NCD associations hold across most of the birthweight range: although the risk of childbirth complications may be greatest among mothers with the smallest birth canals, *any* mother is at risk if she gestates a bigger foetus than she can deliver, resulting in the evolution of a ‘universal defence mechanism’ to reduce obstructed labour risk (**Fig. 4**). Essentially, every foetus is subject to these constraining mechanisms [39], and achieves lower growth than would be optimal for both maximizing its survival and minimizing its NCD risk [6, 24].

Third, although growth faltering often commences *in utero* [133, 134], nutritional interventions during pregnancy have limited efficacy in improving birth size [135]. We argue that the regulatory mechanisms summarized above prevent nutritional supplementation during pregnancy from promoting birth size, unless early foetal growth is substantially impaired. This is consistent with small increases in mean birthweight following supplementation, but substantial reductions in small-for-gestational age births [135]. An alternative potential strategy for public health interventions, to improve foetal growth and lower adult NCD risk, could be to promote girls’ pelvic growth during post-natal life. **Figure 2** indicates that early childhood may be an important window for such interventions, which could align with current efforts to reduce stunting. Although nutritional interventions are often targeted at the first 2 years after birth, there is increasing awareness that height may be able to recover from stunting through early childhood [136–138].

Consistent with that, a secular trend in height among mothers and daughters was shown to extend to dimensions of the pelvis [35]. Moreover, a recent analysis of data from the early 20th century in Switzerland found that average maternal height increased by ~4 cm over 60 birth years, and that average birthweight of their offspring demonstrated an upward trend 28 years later [139]. From the opposite perspective, the fact that maternal height has shown a negligible increase in sub-Saharan Africa and South Asia over the last century [140] may be a major factor constraining birthweight trends in these regions, with implications for diabetes risk [141].

Finally, our approach helps us understand what happens when the regulatory systems that we have proposed do not function effectively. Maternal obesity and T1DM, T2DM or gestational diabetes mellitus present the foetus with the threat of maternal hyperglycaemia. The foetus can defend against this by raising its insulin levels, driving increased fat deposition and macrosomia [142]. Excess neonatal adiposity then becomes another emerging risk pathway for NCDs [143, 144], a scenario assumed to have been rare until recent historical periods. Where appropriate facilities are available, the large birth size associated with macrosomia is typically resolved by caesarean delivery [145, 146], negating the selective pressure of obstructed labour. The risk of obstructed labour associated with macrosomia may explain why foetal fat deposition occurs primarily in the third trimester, and accelerates rapidly after birth [147].

The threat to delivery generated by high foetal adiposity is particularly apparent in mothers of shorter stature, with smaller pelvic dimensions. In India, for example, maternal short stature and overweight/obesity show interactive associations with the risk of caesarean section [126], a pattern replicated in other low- and middle-income countries [148], and consistent with the interactive association of maternal short stature and large neonatal head circumference [36]. Global increases in the demand for caesareans may, therefore, reflect the exacerbation of maternal obesity by the poor linear growth of mothers in early life [126]. If birthed successfully, the macrosomic foetus experiences long-term health penalties in the form of elevated obesity and NCD risk [6]. In an Indian birth cohort, it was women with larger pelvic dimensions and higher BMI whose offspring had higher NCD risk. These offspring were short at birth but had high ponderal index, indicating that their exposure to high maternal fasting plasma glucose had simultaneously constrained their somatic growth yet promoted excess foetal adiposity [149].

Beyond effects on birth size, maternal hyperglycaemia may also have implications for gestation length. A study of mothers with T1DM identified a dose-response association between peri-conceptional levels of glycosylated haemoglobin, a marker of poor glucose control, and the risk of spontaneous preterm birth [150]. Similarly, gestational diabetes mellitus has been associated with a 50% increased risk of preterm birth relative to non-diabetic mothers [151]. A tendency for higher levels of maternal circulating fuel to precipitate earlier delivery would be adaptive for the mother in reducing obstructed labour risk, and also for the offspring if it survived. This suggests that even if high birthweights have become common only recently, they may have occurred sufficiently frequently in ancestral populations for selection to have favoured protective mechanisms that trigger earlier delivery. Nevertheless, high rates of caesarean section among mothers with gestational diabetes [61] indicate that these defence mechanisms have limited efficacy.

## EVOLUTION AND THE DOHaD HYPOTHESIS

Our approach has implications for evolutionary models of the DOHaD hypothesis. The thrifty phenotype hypothesis, which proposed that maternal undernutrition imposes a trade-off on the offspring (early survival vs long-term NCD risk), remains well accepted, though as discussed above, the importance of maternal undernutrition in driving birthweight-NCD associations merits re-evaluation [152]. In the Netherlands, research has supported both components of the hypothesis—that famine raises infant mortality more than foetal mortality, indicating that thrifty foetal growth enables immediate survival [153], and that survivors of *in utero* exposure to famine have elevated NCD risk in adulthood [154, 155], mediated by epigenetic alterations [156]. Both alleles and maternal effects may drive birthweight–NCD associations, though the relative importance of these two mechanisms may vary by outcome [12, 100, 157]. However, the association of birthweight with NCD risk across the birthweight range encompasses large numbers who cannot be considered to have been exposed to overt maternal undernutrition.

The predictive adaptive response hypothesis offered an alternative explanation for birthweight–NCD associations, proposing that metabolic adjustments associated with low birthweight would prove adaptive if the adult remained in energy-scarce environments [17, 158]. Only if the adult environment showed a ‘mismatch’ with foetal experience would NCDs develop [17]. However, whether the foetus can accurately anticipate adult conditions has been questioned [19, 159], and studies have failed to demonstrate improved survival or reproduction among adults in energy-scarce environments who were exposed to foetal malnutrition [20, 21, 160]. Instead, studies on humans and animals tend to support a ‘silver spoon’ hypothesis, whereby offspring that receive greater maternal investment in early life have better adult outcomes, regardless of the adult environment [21, 160, 161].

We offer an alternative evolutionary hypothesis for the birthweight-NCD associations that are central to the DOHaD framework, by addressing both genetic and environmental causes of the association. We shift attention away from short-term ecological stresses and focus on how foetal growth patterns may be shaped by selective pressures related to viable vaginal childbirth. We suggest that foetal growth is strongly shaped by maternal capital [162], a term that summarizes maternal traits that favour investment in the offspring. Small dimensions of the birth canal, for example due to maternal stunting, indicate a decrement in maternal capital. Foetal responses to maternal undernutrition may therefore reflect only one component of a broader system of physiological sensitivity that, together with complementary genetic mechanisms, coordinates foetal growth under the selective pressure of obstructed labour.

We suggest that the coordination systems described above were strongly exposed to selection over hundreds of thousands of years during the evolution of the *Homo* genus, due to increasing neonatal encephalisation. However, a new selective pressure occurred much more recently in the last 10,000 years, whereby the emergence of agriculture exposed populations to diets high in carbohydrate [163]. This relatively brief exposure may have limited the opportunity for selection to respond, resulting in inadequate coordination of foetal growth when mothers achieve high weight or fail to regulate their substrate metabolism. As ‘nutrition transition’ introduces new ultra-processed food products [164], this metabolic stress increasingly impacts women in the global south who already experienced poor growth in early life, contributing to escalating rates of caesareans [165].

## TESTING THE HYPOTHESIS

Our hypothesis assumes that selection has favoured an over-arching regulatory system that regulates foetal growth and gestation in association with maternal metabolism and morphology. To test this hypothesis, several questions merit further investigation.

First, data on the obstetric pelvis could be incorporated in Mendelian Randomization studies examining associations of parental and foetal genotypes with birthweight and NCD risk. If our hypothesis is correct, then alleles associated with maternal height may also explain variability in pelvic dimensions. Moreover, alleles associated with smaller dimensions of the obstetric pelvis would be expected to reduce birth size and gestation length, mediated by maternal metabolic signals. The same alleles may predict adult NCD risk.

Second, markers of maternal growth constraint in early life may predict smaller pelvic dimensions and shorter gestation, as well as lower birthweight in the offspring. Short adult stature has been associated with the risk of preterm birth [166], but specific links of stunting in early life with pelvic dimensions remain to be evaluated. Such associations would indicate the sensitivity of pelvic and foetal growth to environmental drivers of obstructed labour, mediated by maternal growth patterns in early life.

Third, we predict that secular increases in maternal height would drive corresponding increases not only in birthweight but also in gestation length. This hypothesis is difficult to test, as other trends would need to be taken into account, for example, maternal age, BMI, gestational diabetes and smoking, as well as medical practices. In 2020, the global regions with high levels of child stunting (southern Asia and sub-Saharan Africa) accounted for 56% of global live-births but ~65% of all preterm births [167]. Therefore, it is plausible that success in reducing stunting might eventually increase gestation lengths.

## CONCLUSIONS

Nutritional scarcity was likely a key selective pressure throughout human evolution. The theory of ‘parent-offspring conflict’ has been used to understand how mother and foetus compete for scarce metabolic substrates [114]. Here, we highlight a complementary issue that may also have been a crucial selective pressure, by considering how successful vaginal delivery requires defence against excessive nutritional investment in the foetus.

We suggest that a substantial component of variability in birth size represents the outcome of dynamic materno-foetal interactions that evolved to balance foetal growth against childbirth complications. This perspective helps explain both why inverse birthweight-NCD associations are evident across the majority of the range of birthweight, and why interventions intended to increase birthweight through nutritional supplementation have modest impact, as they may simply trigger the defence mechanisms we have described. We do not discount a causal role for maternal undernutrition, infection or pathology in NCD risk, rather, we argue that those stresses may interact with more fundamental regulatory systems for coordinating foetal growth that operate in every mother–foetus dyad.

Our hypothesis offers an evolutionary explanation for the combined genetic and environmental associations of birthweight with NCDs. Any constraint on birthweight is expected, through its effects on homeostatic traits, to undermine protection against NCDs in later life [41]. We hope our approach may interest scientists from diverse fields, including physiology, genetic epidemiology, human evolution and anthropology, and help promote inter-disciplinary research on maternal and child health.

## Supplementary Material

eoae002_suppl_Supplementary_Tables_S1-S3_Figures_S1

## Data Availability

There are no data underlying this work.
