# Supplementary material for: Reconsidering the developmental origins of adult disease paradigm: The ‘metabolic coordination of childbirth’ hypothesis
Source: Evol Med Public Health. 2024 Jan 18;12(1):50–66. doi: 10.1093/emph/eoae002 (PMC10878253; doi:10.1093/emph/eoae002)
Supplement: eoae002_suppl_Supplementary_Tables_S1-S3_Figures_S1 [file eoae002_suppl_supplementary_tables_s1-s3_figures_s1.pdf]

Reconsidering the developmental origins of disease paradigm: the 'metabolic coordination of childbirth' hypothesis

Table S1. Heritability of birth weight

Population	Sample size	Method	Heritability	Ref
Sweden	2009 twin pairs	Quantitative genetic methods	42%	1.
Norway	67,795 family trios	Pearson correlation	25%	2.
Norway	101,748 mother-offspring pairs	Path analysis	31%	3.
Netherlands	3407 singletons, 33694, twins	Genetic structural equation modelling	26% singletons, 29% twins	4.
UK	110 child twin pairs	Quantitative genetic methods	44%	5.
US	148 twin pairs	Genetic structural equation modelling	66%*	6.
Australia, Netherlands, US	2679 twin pairs	Quantitative genetic methods	11-12%	7.
Japan, South Korea	2334 twin pairs	Quantitative genetic methods	15-17%	7.
Iran, Malaysia	430 twin pairs	Genetic structural equation modelling	53%	8.

* Estimated fetal weight at 38 weeks

1. Clausson B, Lichtenstein P, Cnattingius S. Genetic influence on birthweight and gestational length determined by studies in offspring of twins. BJOG Int J Obstet Gynaecol. 2000; 107, 375–381.
2. Magnus P, Gjessing HK, Skrondal A, Skjærven R. Paternal contribution to birth weight. J Epidemiol Community Health 2001;55:873–877
3. Lunde A, Melve KK, Gjessing HK, Skjaerven R, Irgens LM. Genetic and environmental influences on birth weight, birth length, head circumference, and gestational age by use of population-based parent-offspring data. Am J Epidemiol. 2007; 165, 734–741.
4. Mook-Kanamori DO, van Beijsterveldt CE, Steegers EA, Aulchenko YS, Raat H, Hofman A, Eilers PH, Boomsma DI, Jaddoe VW. Heritability estimates of body size in fetal life and early childhood. PLoS One. 2012;7(7):e39901.
5. Beardsall K, Ong KK, Murphy N, Ahmed ML, Zhao JH, Peeters MW, Dunger DB. Heritability of childhood weight gain from birth and risk markers for adult metabolic disease in prepubertal twins. J Clin Endocrinol Metab. 2009 Oct;94(10):3708-13.
6. Workalemahu T, Grantz KL, Grewal J, Zhang C, Louis GMB, Tekola-Ayele F. Genetic and Environmental Influences on Fetal Growth Vary during Sensitive Periods in Pregnancy. Sci Rep. 2018 May 8;8(1):7274.

7. Hur YM, Luciano M, Martin NG, Boomsma DI, Iacono WG, McGue M, Shin JS, Jun JK, Ooki S, van Beijsterveldt CE, Han JY. A comparison of twin birthweight data from Australia, the Netherlands, the United States, Japan, and South Korea: are genetic and environmental variations in birthweight similar in Caucasians and East Asians? Twin Res Hum Genet. 2005 Dec;8(6):638-48.
8. Jahanfar S. Birth weight and anthropometric measurements of twins. Ann Hum Biol. 2018 Aug;45(5):395-400.

Table S2. Associations of maternal pelvic dimensions with maternal height and offspring birth weight and NCD outcomes in historical cohorts

Setting	Birth period	N*	Follow-up	Maternal height	Birth size	Stroke risk	Ref
Hertfordshire and Sheffield, UK	1907-1930	13,249 M	-		Conjugate, intercristal, and interspinal diameters correlated with birth weight (r=0·27, 0·24, and 0·15, respectively). Birth weight for flat/not 3.13 vs 3.38 kg, difference 245 g	SMR for stroke 184 (CI 67–396) for flat pelvis, 104 (CI 78–138) for remainder	1
Mysore, India	1934-1953	241 MF	1976-1997			Pulse wave velocity higher in those whose mothers had smaller external conjugate diameters	2.
Helsinki, Finland	1924-1933	3,639 M	1971-1995	81% of mothers with flat pelvis below-average height (1.58 m)	Flat pelvis associated with increased ratio of head circumference to birth weight	HR for admissions/deaths for stroke 1.5 (CI 1.1, 2.1) per kg decrease in birth weight adjusted for head circumference	3.
Helsinki, Finland	1934-1944	7,960 MF	1971-2003	Mothers with conjugate diameters of ≤18 cm on average 4.1 cm shorter	Offspring of women with conjugate diameters of ≤18 cm were 0.4 cm shorter, 146 g lighter, 0.3 kg/m^2 lower body mass index at birth (P<0.0001 for each)	Conjugate diameter ≤18 cm increased risk of stroke (HR 1.62, CI, 1.30 to 2.02). Findings similar for hemorrhagic and thrombotic stroke. Intercristal and interspinous diameters had null associations	4.
Uppsala, Sweden	1915-1929	6,362 MF	1964-2008		Offspring of mothers with flat pelvis had lower birth weight (difference 145 g, CI 21, 219)	Flat pelvis increased risk of thrombotic stroke in F (HR 1.66, CI 1.09, 2.54) but not M (0.90, CI 0.53, 1.53). Conjugate diameter ≤18 cm, relative to >19 cm, increased risk of haemorrhagic stroke in M (HR 1.76, CI 1.07, 2.90) but not F (HR 1.39, CI 0.70, 2.40)	5.

NCD – noncommunicable disease. M – male, F – female. SMR – standardised mortality ratio. HR – hazard ratio. CI = 95% confidence interval
* Sample size refers where relevant to subsample with maternal pelvic measurements

1. Martyn VN, Barker DJ, Osmond C. Mothers' pelvic size, fetal growth, and death from stroke and coronary heart disease in men in the UK. Lancet 1996; 348, 1264-1268.
2. Kumaran K, Fall CH, Martyn CN, Vijayakumar M, Stein C, Shier R. Blood pressure, arterial compliance, and left ventricular mass: no relation to small size at birth in south Indian adults. Heart 2000; 83, 272-277.
3. Eriksson JG, Forsen T, Tuomilehto J, Osmond C, Barker DJ. Early growth, adult income, and risk of stroke. Stroke 2000; 31, 869-874.
4. Osmond C, Kajantie E, Forsen TJ, Eriksson JG, Barker DJ. Infant growth and stroke in adult life: the Helsinki birth cohort study. Stroke 2007; 38, 264-270.
5. Heshmati A, Chaparro MP, Koupil I. Maternal pelvic size, fetal growth and risk of stroke in adult offspring in a large Swedish cohort. J Dev Orig Health Dis 2016; 7, 108-113.

Table S3. Epidemiological evidence for association of maternal height with gestational age or risk of preterm birth

Population	Sample size	Method	Association	Ref
Sweden	192,432	Birth register study	Every cm decrease in maternal stature was associated with 0.2 days shortening of gestational age in the offspring ($p<0.0001$)	1.
Norway	3,497	Population-based prospective study	Pregnancy length increased with maternal height. Estimating date of delivery from ultrasound, mothers in lower height quintile (<163 cm had 4.3 days less gestation relative to those in upper quintile (≥ 173 cm)	2.
9 studies		Meta-analysis	Increased unadjusted risk of preterm birth in short-statured women compared to women of reference height (RR 1.23,95% CI 1.11, 1.37)	3.
12 LMICs*	36,803	Prospective cohort studies	All height categories below reference associated with risk of term SGA, preterm AGA, and preterm SGA births (compared with term AGA), with women <145 cm having highest RR (term SGA aRR: 2.03; 95% CI: 1.76, 2.35; $P < 0.001$; preterm AGA aRR: 1.44; 95% CI: 1.26, 1.66; $P = 0.011$; preterm SGA—aRR: 2.13; 95% CI: 1.42, 3.21; $P = 0.031$)	4.
Bangladesh	2,655	Population-based cohort	Maternal short stature <145 cm associated with preterm AGA (RRR 1.45, 95% CI 1.02, 2.05; $p < 0.05$), and preterm SGA (RRR 14.40, 95% CI 1.82, 113.85; $p < 0.05$)	5.

LMIC – low-/middle-income country; AGA – appropriate for gestational age; SGA - small for gestational age.

1. Derraik JGB, Lundgren M, Cutfield WS, Ahlsson F (2016) Maternal Height and Preterm Birth: A Study on 192,432 Swedish Women. PLoS ONE 11(4): e0154304.
2. Myklestad K, Vatten LJ, Magnussen EB, Salvesen KÅ, Romundstad PR. Do parental heights influence pregnancy length?: A population-based prospective study, HUNT 2. BMC Pregnancy Childbirth. 2013 Feb 5;13:33.
3. Han Z, Lutsiv O, Mulla S, McDonald SD; Knowledge Synthesis Group. Maternal height and the risk of preterm birth and low birth weight: a systematic review and meta-analyses. J Obstet Gynaecol Can. 2012 Aug;34(8):721-746.
4. Kozuki N, Katz J, Lee AC, Vogel JP, Silveira MF, Sania A, Stevens GA, Cousens S, Caulfield LE, Christian P, Huybregts L, Roberfroid D, Schmiegelow C, Adair LS, Barros FC, Cowan M, Fawzi W, Kolsteren P, Merialdi M, Mongkolchati A, Saville N, Victora CG, Bhutta ZA, Blencowe H, Ezzati M, Lawn JE, Black RE; Child Health Epidemiology Reference Group Small-for-Gestational-Age/Preterm Birth Working

Group. Short Maternal Stature Increases Risk of Small-for-Gestational-Age and Preterm Births in Low- and Middle-Income Countries: Individual Participant Data Meta-Analysis and Population Attributable Fraction. J Nutr. 2015 Nov;145(11):2542-50.

5. Khanam R, Lee AC, Mitra DK, Ram M, Das Gupta S, Quaiyum A, Choudhury A, Christian P, Mullany LC, Baqui AH. Maternal short stature and under-weight status are independent risk factors for preterm birth and small for gestational age in rural Bangladesh. Eur J Clin Nutr. 2019 May;73(5):733-742.

Figure S1. Heritability of birth weight and length through pregnancy and infancy in the Netherlands. For both components of growth, heritability is markedly lower before birth compared to mid-infancy onwards.

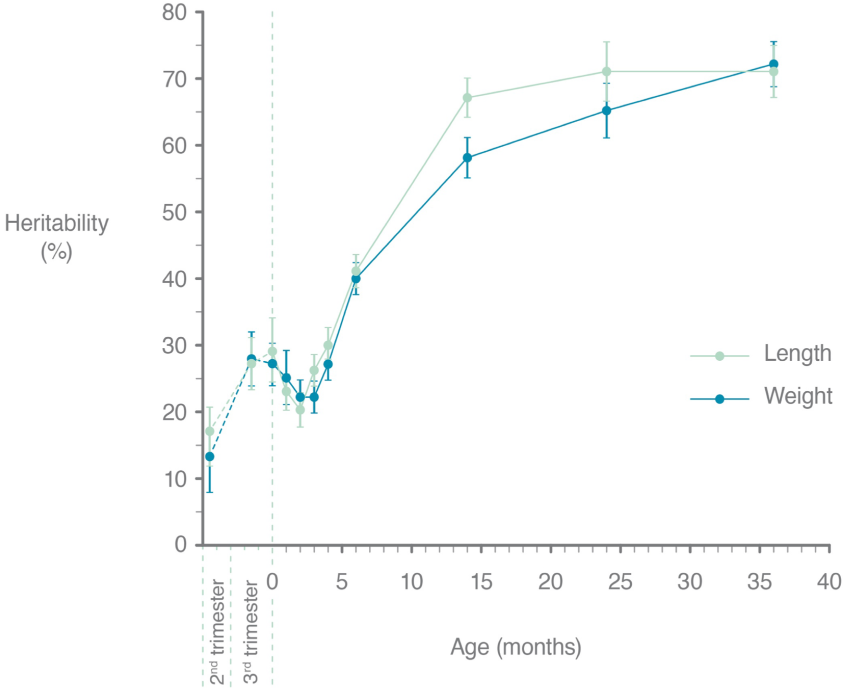

Based on data from Mook-Kanamori DO, van Beijsterveldt CE, Steegers EA, et al.; Heritability estimates of body size in fetal life and early childhood. PLoS One 2012;7(7):e39901. doi: 10.1371/journal.pone.0039901.
